# Effects of surgical management for gastrointestinal stromal tumor patients with liver metastasis on survival outcomes

**DOI:** 10.3389/fonc.2024.1289885

**Published:** 2024-01-29

**Authors:** Lei Liu, Xiaomin Xia, Yiheng Ju, Simeng Zhang, Ning Shi, Yongxing Du, Hanxiang Zhan, Shanglong Liu

**Affiliations:** ^1^Department of Gastrointestinal Surgery, Affiliated Hospital of Qingdao University, Qingdao, China; ^2^Department of Prosthodontics, Affiliated Hospital of Qingdao University, Qingdao, China; ^3^Department of General Surgery, Guangdong Provincial People’s Hospital (Guangdong Academy of Medical Sciences), Southern Medical University, Guangzhou, China; ^4^Department of Pancreatic and Gastric Surgery, National Cancer Center/Cancer Hospital, Chinese Academy of Medical Sciences and Peking Union Medical College, Beijing, China; ^5^Department of General Surgery, Qilu Hospital, Shandong University, Jinan, Shandong, China

**Keywords:** gastrointestinal stromal tumor, synchronous liver metastasis, surgery, nomograms, survival

## Abstract

**Purpose:**

To investigate the effect of surgical resection on survival in gastrointestinal stromal tumors synchronous liver metastasis (GIST-SLM) and to develop clinically usable predictive models for overall survival (OS) and cancer-specific survival (CSS) in patients.

**Methods:**

We identified patients in the SEER database diagnosed with GISTs from 2010 to 2019. We used propensity score matching (PSM) to balance the bias between the Surgery and No surgery groups. Kaplan-Meier(K-M) analysis was used to detect differences in OS and CSS between the two groups. The nomogram to predict 1, 3, and 5-year OS and CSS were developed and evaluated.

**Results:**

After PSM, 228 patients were included in this study. There were significant differences in 1, 3, and 5-year OS and CSS between the two groups (OS: 93.5% vs. 84.4%, 73.2% vs. 55.3%, 60.9% vs. 36.9%, P=0.014; CSS: 3.5% vs.86.2%,75.3% vs.57.9%, 62.6% vs. 42.9%, P=0.02). We also found that patients who received surgery combined with targeted therapy had better OS and CSS at 1, 3, and 5 years than those who received surgery only (OS: 96.6% vs.90.9%, 74.9% vs. 56.8%, 61.7% vs. 35.5%, P=0.022; CSS: 96.6% vs. 92.1%, 77.4% vs.59.2%,63.8% vs. 42.0%, P=0.023). The area under the curve (AUC) was 0.774, 0.737, and 0.741 for 1, 3, and 5-year OS, respectively, with 0.782 and 0.742 for 1, 3, and 5-year CSS. In the model, C-index was 0.703 for OS and 0.705 for CSS and showed good consistency.

**Conclusion:**

Surgical treatment can improve the OS and CSS of patients with GIST-SLM. In addition, the combination with chemotherapy may be more favorable for the long-term survival of patients. Meanwhile, we constructed the nomograms for predicting OS and CSS at 1, 3, and 5-year, and validated them internally. Our model can contribute to clinical management and treatment strategy optimization.

## Introduction

Gastrointestinal stromal tumors (GISTs) are the most common subtype of soft tissue tumors originating from the interstitial cells of Cajal (ICC) and have a wide range of tumor characteristics, from small lesions with benign behavior to aggressive sarcomas (1). The incidence of GIST is 10-15 cases per million worldwide, which can occur anywhere in the gastrointestinal tract, but is most common in the stomach (50-60%) and small intestine (30-35%), as well as in the colon and rectum (5%) and esophagus (1%) (2). Approximately 11-47% of GIST have metastases at the time of discovery. The most common site of metastasis is the liver, and it may also occur in the peritoneum, lungs, brain, and bone (3, 4). GIST patients with distant metastases have a poorer prognosis, with a median survival time of about 20 months, which is much lower than that of patients without distant metastases (3). The study by Yang et al. showed that the median survival times of GIST patients initially diagnosed with liver, bone, and lung metastases were 49, 18, and 20 months, respectively (5).

Before 2000, patients with metastatic GIST were mainly treated with surgical resection or chemotherapy, but the patients’ response to chemotherapy was poor, with an effective rate of less than 10%, and the prognosis after surgical resection of liver metastases was also poor with a 5-year overall survival rate of 30% only (6, 7). GIST is an acquired function mutation of c-KIT and platelet-derived growth factor receptor alpha (PDGFRA), so the advent of tyrosine kinase inhibitors (TKIs) has revolutionized the treatment regimen for GISTs (8, 9). Imatinib mesylate is an orally available, selective small-molecule competitive inhibitor of tyrosine kinases such as KIT and PDGFRA, which is a highly effective targeted agent for the treatment of patients with advanced GISTs and has become the basic therapy for metastatic GISTs (10, 11). However, it remains controversial whether surgical resection is beneficial for the long-term survival of gastrointestinal stromal tumors synchronous liver metastasis (GIST-SLM) (1).

To the best of our knowledge, there are some articles on GIST-SLM. However, the amount of data is small and the baseline data is unbalanced, it lacks representativeness (12–14). While related studies in the SEER database analyzed the effect of surgical treatment on the prognosis of patients with liver metastatic gastrointestinal mesenchymal stromal tumors, they all included patients with metastases from other sites (15, 16). Therefore, it is necessary to find reliable clinical methods to predict the prognosis of patients with GIST-SLM and provide rational interventions to improve it. Among the available clinical decision-making tools, the nomogram is the most accurate and discriminating method for predicting the prognosis of cancer patients, and it may be one of the most valuable tools in precision medicine, so it can be used to predict overall survival (OS) and cancer-specific survival (CSS) in GIST-SLM patients.

On this basis, we select data from the SEER database of patients diagnosed with GIST from 2010 to 2019 and screened GIST-SLM patients. The primary aim of this study is to investigate the effect of surgical resection on OS and CSS in patients with GIST-SLM. And it also aims to determine the factors influencing survival time in GIST-SLM patients and to develop clinically usable predictive models for overall survival (OS) and cancer-specific survival (CSS) in patients with GIST-SLMs.

## Materials and methods

### Data source and patient selection

**Figure 1:**
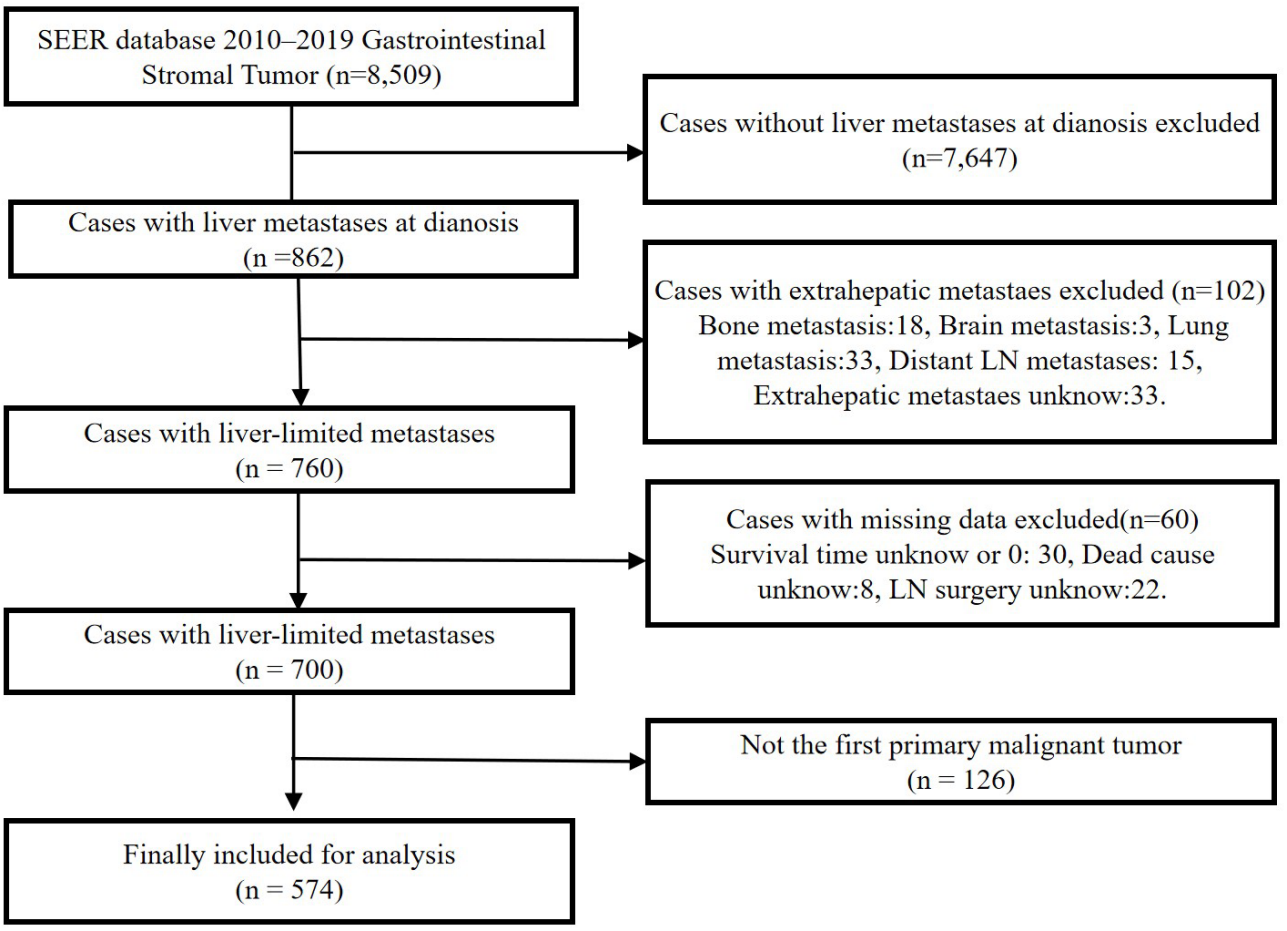
Patient inclusion and exclusion flow chart.

We identified patients in the SEER database diagnosed with GISTs from 2010 to 2019 for retrospective analysis. SEER is a population-based cancer registry with 18 sites that cover approximately 27.8% of the United States (17). The SEER data record includes the patients’ registration number, personal information, site of the primary lesion, tumor size, tumor code, treatment, and cause of death. Patients with GISTs were identified using a specific histologic code (International Classification of Diseases for Oncology [ICD-O] code 8936). Patient selection is outlined in **Figure 1**. Ethical approval and informed consent were exempted by the ethics committee on account of the public availability of all the data in SEER database.

Patients were stratified by age of younger (<65 years old) and older (≥65 years old).

Race was grouped as black, white, or some other race (such as Asian/Pacific Islander and American India/AKNative) and unavailable. ICD-O site was used for identifying tumor sites, which were categorized as the stomach, small intestine, and other organs. The grade was grouped as poor differentiated or undifferentiated, well or moderately differentiated, and unknown. Tumor size was grouped as <5.0 cm, 6-10 cm, >10 cm, and unknown. The local lymph node metastasis was grouped as yes, no, and unknown. Chemotherapy was grouped as yes and no. We defined a mitotic index variable as low (≤5mitoses per high-power field), high (>5mitoses per high-power field), and unknown. Marital status was classified as married (consisting of common law), unmarried (including widowed, single, domestic partner, divorced, and separated). Overall survival (OS) and cancer-specific survival (CSS) were utilized as the primary outcomes.

### Statistical analysis

To investigate the impact of surgery on OS and CSS in GIST-SLM patients, we categorized patients into Surgery and No Surgery groups. Chi-square tests were performed for the comparison of baseline factors for categorical variables. Because this study was retrospective, we initially compared the baseline data of the two groups and found bias in Age, Race, Site, Grade, Tumor size, LN Metastasis, LN surgery, Mitotic count, and Marital status. To balance the bias between the Surgery and No surgery groups, we used 1:1 propensity score matching (PSM) with 0.2 caliper width. Kaplan-Meier(K-M) analysis and log-rank test were used to detect differences in OS and CSS between the Surgery and No surgery groups.

Univariate and multivariate Cox proportional hazards regression analyses were performed to determine the hazard ratio (HR) and 95% confidence interval (CI). To improve the predictive power and interpretability of the model, a stepwise regression method (both directions) was used for variable screening under the Akaike information criterion (AIC). A nomogram to predict 1, 3, and 5-year OS and CSS was developed based on independent prognostic factors. We evaluated the reliability and accuracy of the predictive model through the receiver operating characteristic (ROC) curve, area under the curve (AUC), concordance index (C-index), and calibration curve. Decision curve analysis (DCA) is a method to evaluate the practical value of the predictive model by estimating the net benefit under different risk thresholds. These evaluations were also applied to the internal validation set. (A bootstrapped resample with 100 iterations from the training set for validation). All analyses were performed using R version 4.3.0 (The R Foundation for Statistical Computing, Vienna, Austria). P-values < 0.05 on both sides were considered statistically significant.

## Results

### Entire cohort characteristics

A total of 8,509 patients diagnosed with GIST from 2010 to 2019 were screened from the SEER database. After eliminating 7,935 patients based on the exclusion criteria, 574 patients with GIST with synchronous liver metastases (GIST-SLM) were included for further analysis (**Figure 1**). To investigate the impact of surgery on OS and CSS in patients, we categorized patients into Surgery group (n=213) and No surgery group (n=361). The basic characteristics of the two groups before and after PSM are shown in **Table 1**.

**Table 1:** Comparison of the characteristics of all patients.

Characteristic	Before PSM		P	After PSM		P
	Surgery group (n=213)	No surgery group (n=361)		Surgery group (n=114)	No surgery group (n=114)	
Age			<0.001			0.587
<65 ≥65	142 (66.7) 71 (33.3)	181 (50.1) 180 (49.9)		67 (58.8) 47 (41.2)	72 (63.2) 42 (36.8)	
Sex			0.533			0.688
Male Female	128 (60.1) 85 (39.9)	206 (57.1) 155 (42.9)		63 (55.3) 51 (44.7)	67 (58.8) 47 (41.2)	
Race			<0.001			0.975
White Black Other	148 (69.5) 33 (15.5) 32 (15.0)	218 (60.4) 53 (14.7) 90 (24.9)		74 (64.9) 23 (20.2) 17 (14.9)	74 (64.9) 24 (21.1) 16 (14.0)	
Site			<0.001			0.707
Stomach Small intestine Other	103 (48.4) 83 (39.0) 27 (12.7)	224 (62.0) 55 (15.2) 82 (22.7)		67 (58.8) 29 (25.4) 18 (15.8)	73 (64.0) 26 (22.8) 15 (13.2)	
Grade			<0.001			0.556
Well/moderate Poor/un Blank	45 (21.1) 46 (21.6) 122 (57.3)	14 (3.9) 17 (4.7) 330 (91.4)		13 (11.4) 18 (15.8) 83 (72.8)	10 (8.8) 14 (12.3) 90 (78.9)	
Turmor size (cm)			<0.001			0.879
≤5 6-10 >10 Blank	22 (10.3) 69 (32.4) 104 (48.8) 18 (8.5)	62 (17.2) 81 (22.4) 108 (29.9) 110 (30.5)		14 (12.3) 31 (27.2) 53 (46.5) 16 (14.0)	16 (14.0) 27 (23.7) 52 (45.6) 19 (16.7)	
LN Metastases			<0.001			0.398
No Yes Blank	266 (73.7) 37 (10.2) 58 (16.1)	182 (85.4) 24 (11.3) 7 (3.3)		93 (81.6) 15 (13.2) 6 (5.3)	100 (87.7) 9 (7.9) 5 (4.4)	
LN surgery			<0.001			<0.001
No Yes	126 (59.2) 87 (40.8)	359 (99.4) 2 (0.6)		63 (55.3) 51 (44.7)	114 (100.0) 0 (0.0)	
Mitotic count (mitoses/50 HPFs)			<0.001			0.845
≤5 >5 Unknown	77 (36.2) 48 (22.5) 88 (41.3)	31 (8.6) 14 (3.9) 316 (87.5)		27 (23.7) 14 (12.3) 73 (64.0)	25 (21.9) 12 (10.5) 77 (67.5)	
Marital status			0.054			0.171
Married Unmarried	134 (62.9) 79 (37.1)	196 (54.3) 165 (45.7)		66 (57.9) 48 (42.1)	77 (67.5) 37 (32.5)	
Chemotherapy			0.905			1.000
No Yes	173 (81.2) 40 (18.8)	296 (82.0) 65 (18.0)		20 (17.5) 94 (82.5)	20 (17.5) 94 (82.5)	

Before PSM, in the Surgery group, the proportion of ≥65 years and sites in the stomach was significantly less than in the No surgery group (33.3% vs. 49.9%, P<0.001 and 48.4% vs. 62.0%, P<0.001). Two groups had almost equal percentages of males (60.1% vs. 57.1%, P=0.533) and chemotherapy patients (18.8% vs. 18.0%, P=0.905). Compared No surgery group, surgery group shows more proportion of Caucasian person (69.4% vs. 60.4%, P<0.001), Well/moderate differentiation patients (21.1% vs. 3.9%, P<0.001), Tumor size >10cm (48.8% vs. 29.9%, P<0.001), LN surgery patients (40.8% vs. 0.6%, P<0.001), mitotic count ≤ 5 mitoses/50 HPFs (36.2% vs. 8.6%, P<0.001) and married patients (62.9% vs. 54.3%, P=0.054). In addition, the percentage of patients with lymph node metastasis was higher in the Surgery group than in the No Surgery group (85.4% vs. 73.7%, P < 0.001).

### Impact of surgery on OS and CSS of patients

**Figure 2:**
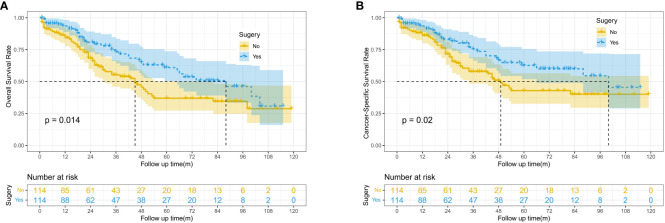
Kaplan-Meier survival curves for OS and CSS between the surgery and no surgery groups; **(A)** Overall survival (OS). **(B)** Cancer-specific survival (CSS).

After PSM, 228 patients were included in this study, with 114 patients in both the surgery and no-surgery groups. Since lymph node dissection was performed during surgery, we think that the baseline data of the two groups were balanced. The results showed that the median OS and CSS of patients were higher in the Surgery group than in the No surgery group (88 vs. 45 months, 100 vs. 49 months). There were significant differences in 1, 3 and 5-year OS between two groups (93.5% vs. 84.4%, 73.2% vs. 55.3%, 60.9% vs. 36.9%, P=0.014) (**Figure 2A**). Similarly, 1, 3 and 5-year CSS was higher in the surgery group than in the no surgery group (93.5% vs.86.2%,75.3% vs.57.9%, 62.6% vs. 42.9%, P=0.02) (**Figure 2B**). As shown in **Table 2**, we performed subgroup analyses based on whether or not chemotherapy. The chemotherapy group had a greater proportion of patients aged <65 years and more patients with tumor sizes >10cm. This suggests that younger patients with larger tumors were more likely to have received subsequent targeted therapy.

**Table 2:** Subgroup analysis of chemotherapy.

Characteristic	Chemotheraphy group (n=188)	No Chemotherapy group (n=40)	P
Age			0.036
<65 ≥65	121 (64.4) 67 (35.6)	18 (45.0) 22 (55.0)	
Sex			0.807
Male Female	82 (43.6) 106 (56.4)	16 (40.0) 24 (60.0)	
Race			0.340
White Black Other	126 (67.0) 36 (19.1) 26 (13.8)	22 (55.0) 11 (27.5) 7 (17.7)	
Site			0.682
Stomach Small intestine Other	113 (60.1) 36 (19.1) 26 (13.8)	27 (67.5) 5 (12.5) 8 (20.0)	
Grade			0.518
Well/moderate Poor/un Unknown	17 (9.0) 27 (14.4) 144 (76.6)	6 (15.0) 5 (12.5) 29 (72.5)	
Turmor size (cm)			0.019
≤5 6-10 >10 Unknown	21 (11.2) 95 (50.5) 46 (24.5) 26 (13.8)	9 (22.5) 10 (25.0) 12 (30.0) 9 (22.5)	
LN Metastases			0.740
No Yes Unknown	158 (84.0) 20 (10.6) 10 (5.3)	35 (87.5) 4 (10.0) 1 (2.5)	
Surgery			1.000
No Yes	94 (50.0) 94 (50.0)	20 (50.0) 20 (50.0)	
Mitotic count (mitoses/50 HPFs)			0.638
≤5 >5 Unknown	42 (22.3) 20 (10.6) 126 (67.0)	10 (25.0) 6 (15.0) 24 (60.0)	
Maritalstatus			0.099
Married Unmarried	123 (65.4) 65 (34.6)	20 (50.0) 20 (50.0)	
LN surgery			0.023
No Yes	140 (74.5) 48 (25.5)	37 (92.5) 3 (7.5)	

**Figure 3:**
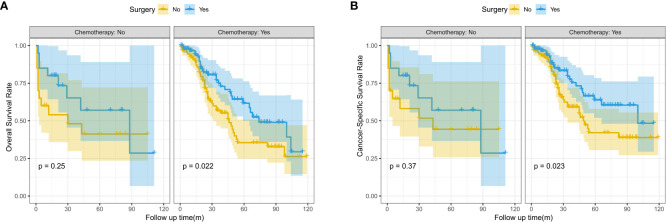
Kaplan-Meier survival curves for OS and CSS between the surgery and no surgery groups stratified by chemotherapy; **(A)** Overall survival (OS); **(B)** Cancer-specific survival (CSS).

In the subgroup survival analysis, we found that patients who received surgery combined with targeted therapy had better OS and CSS at 1, 3, and 5 years than those who received surgery only (OS: 96.6% vs.90.9%, 74.9% vs. 56.8%, 61.7% vs. 35.5%, P=0.022; CSS:96.6% vs. 92.1%, 77.4% vs.59.2%,63.8% vs. 42.0%, P=0.023) (**Figure 3**).

### Prognostic factors and entire cohort survival outcomes

The median follow-time of the entire cohort was 29 months. The 1, 3, and 5-year OS was 84.7, 62.7 and 45.7%, respectively. In addition, the 1, 3, and 5-year CSS was 86.8, 66.5 and 51.7%, respectively. We performed the univariate and multivariate Cox regression analysis of the 574 patients to identify the independent OS and CSS-related risk factors which included Age (OS: HR = 1.85, 95%CI = 1.43–2.38, P <0.001; CSS: HR = 1.58, 95%CI = 1.20-2.09, P=0.001), Sex (OS : HR = 1.32, 95%CI = 1.02–1.71, P = 0.036; CSS: HR = 1.39, 95%CI = 1.04-1.84, P=0.024), Site (OS: HR = 1.69, 95%CI = 1.13–2.55, P = 0.011; CSS: HR = 1.73, 95%CI = 1.10-2.70, P=0.017),Grade (OS: HR = 2.03, 95%CI = 1.20–3.44, P = 0.009; CSS: HR = 2.14, 95%CI = 1.21-3.78, P=0.009),Tumor size (OS: HR = 1.51, 95%CI = 0.99–2.30, P = 0.054; CSS: HR = 1.74, 95%CI = 1.07-2.85, P=0.026), LN metastases (OS: HR = 1.39, 95%CI = 0.96–2.02, P = 0.083; CSS: HR = 1.45, 95%CI = 0.97-2.16, P=0.070), Chemotherapy (OS: HR = 0.73, 95%CI = 0.53–0.99, P = 0.045; CSS: HR = 0.73, 95%CI = 0.52-1.04, P=0.078), Surgery (OS: HR = 0.50, 95%CI = 0.36–0.69, P <0.001; CSS: HR = 0.47, 95%CI = 0.33-0.67, P <0.001) and Marital status (OS: HR = 0.68, 95%CI = 0.53–0.88, P = 0.003; CSS: HR = 0.65, 95%CI = 0.49-0.85, P = 0.002). (**Table 3**; **Figure A1**).

**Table 3:** Univariate and multivariate analysis of OS and CSS.

Variable	OS					CSS			
	Univariate analysis		Multivariate analysis			Univariate analysis		Multivariate analysis	
	HR (95% CI)	P	HR (95% CI)	P		HR (95% CI)	P	HR (95% CI)	P
Age									
<65	Ref		Ref			Ref		Ref	
≥65	2.07 (1.62-1.63)	<0.001	1.85 (1.43-2.38)	<0.001		1.77 (1.36-2.31)	<0.001	1.58 (1.20-2.09)	0.001
Sex									
Female	Ref		Ref			Ref		Ref	
Male	1.10 (0.86-1.40)	0.467	1.32 (1.02-1.71)	0.036		1.14 (0.87-1.49)	0.344	1.39 (1.04-1.84)	0.024
Site									
Stomach	Ref		Ref			Ref		Ref	
Small intestine	0.77 (0.56-1.05)	0.101	1.06 (0.76-1.46)	0.742		0.73 (0.52-1.04)	0.080	1.01 (0.70-1.45)	0.974
Other	1.47 (1.09-1.97)	0.011	1.69 (1.13-2.55)	0.011		1.49 (1.08-2.05)	0.014	1.73 (1.10-2.70)	0.017
Grade									
Well/moderate	Ref		Ref			Ref		Ref	
Poor	1.75 (1.04-2.93)	0.034	2.03 (1.20-3.44)	0.009		1.75 (1.01-3.06)	0.048	2.14 (1.21-3.78)	0.009
Unknown	1.45 (0.93-2.25)	0.100	1.01 (0.63-1.62)	0.966		1.39 (0.87-2.24)	0.169	0.98 (0.59-1.64)	0.943
Tumor size									
≤5	Ref		Ref			Ref		Ref	
6-10	0.92 (0.60-1.42)	0.719	0.92 (0.59-1.43)	0.719		1.15 (0.70-1.89)	0.570	1.14 (0.69-1.89)	0.615
>10	1.47 (0.99-2.19)	0.053	1.51 (0.99-2.30)	0.054		1.72 (1.08-2.73)	0.022	1.74 (1.07-2.85)	0.026
Unknown	2.01 (1.33-3.02)	<0.001	1.64 (1.08-2.50)	0.021		2.53 (1.58-4.07)	<0.001	2.06 (1.27-3.34)	0.004
LN Metastases									
No	Ref		Ref			Ref		Ref	
Yes	1.26 (0.88-1.82)	0.209	1.39 (0.96-2.02)	0.083		1.36 (0.92-2.01)	0.119	1.45 (0.97-2.16)	0.007
Unknown	1.52 (1.07-2.18)	0.020	0.77 (0.46-1.28)	0.313		1.64 (1.12-2.40)	0.010	0.77 (0.44-1.33)	0.346
Chemotherapy									
No	Ref		Ref			Ref		Ref	
Yes	0.68 (0.51-0.91)	0.009	0.73 (0.53-0.99)	0.045		0.70 (0.50-0.96)	0.027	0.73 (0.52-1.04)	0.078
Surgery									
No	Ref		Ref			Ref		Ref	
Yes	0.51 (0.39-0.67)	<0.001	0.50 (0.36-0.69)	<0.001		0.50 (0.37-0.67)	<0.001	0.47 (0.33-0.67)	<0.001
Mitotic count									
≤5	Ref		Ref			Ref		Ref	
>5	1.19 (0.76-1.87)	0.449	(0.87 (0.54-1.41)	0.574		1.07 (0.65-1.77)	0.787	0.79 (0.46-1.34)	0.374
Unknown	1.63 (1.19-2.25)	0.002	1.08 (0.73-1.61)	0.685		1.59 (1.12-2.24)	0.008	1.04 (0.68-1.60)	0.862
Maritalstatus									
No	Ref		Ref			Ref			
Yes	0.66 (0.52-0.84)	<0.001	0.68 (0.53-0.88)	0.003		0.61 (0.47-0.79)	<0.001	0.65 (0.49-0.85)	0.002
LN surgery									
No	Ref		Ref			Ref		Ref	
Yes	0.65 (0.45-0.93)	0.018	1.01 (0.65-1.55)	0.970		0.65 (0.45-0.93)	0.018	0.92 (0.56-1.50)	0.736

### Development and validation of prognostic nomogram for OS and CSS

**Figure 4:**
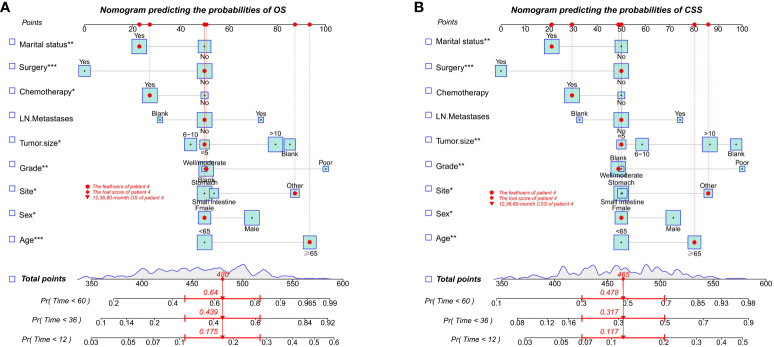
Nomogram predicting the probabilities of survival. **(A)** Overall survival (OS); **(B)** Cancer-specific survival (CSS). * P ≤ 0.05, ** P ≤ 0.01, *** P ≤ 0.001.

**Figure 5:**
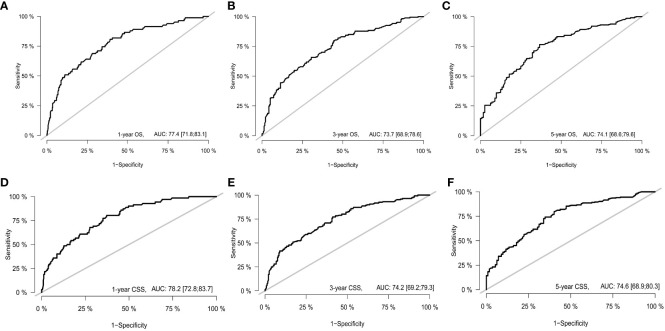
The receiver operating characteristic curve. **(A)** 1-year OS; **(B)** 3-year OS; **(C)** 5-year OS; **(D)** 1-year CSS; **(E)** 3-year CSS; **(F)** 5-year CSS.

**Figure 6:**
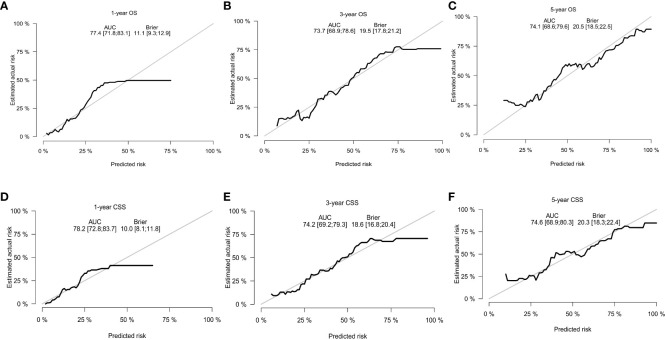
The calibration curves. **(A)** 1-year OS; **(B)** 3-year OS; **(C)** 5-year OS; **(D)** 1-year CSS; **(E)** 3-year CSS; **(F)** 5-year CSS.

With the visualization tool provided by the nomogram, we used the Cox regression model to predict OS and CSS by months of survival of lmGIST patients. The nomogram model is shown in **Figure 4**. In the Cox regression model, C-index was 0.703 for OS and 0.705 for CSS. We tested the nomogram by receiver operating characteristic (ROC) curves in the entire cohort. The area under the curve (AUC) was 0.774(95%CI = 0.718–0.831), 0.737(95%CI = 0.689–0.786), and 0.741(95%CI = 0.686–0.796) for 1, 3 and 5-year OS, respectively, with 0.782(95%CI = 0.728–0.837) and 0.742(95%CI = 0.692–0.793) for 1, 3 and 5-year CSS (**Figure 5**). **Figure 6** showed the calibration curves of the nomogram. These indicated that the new prediction model had a great performance for OS and CSS.

**Figure 7:**
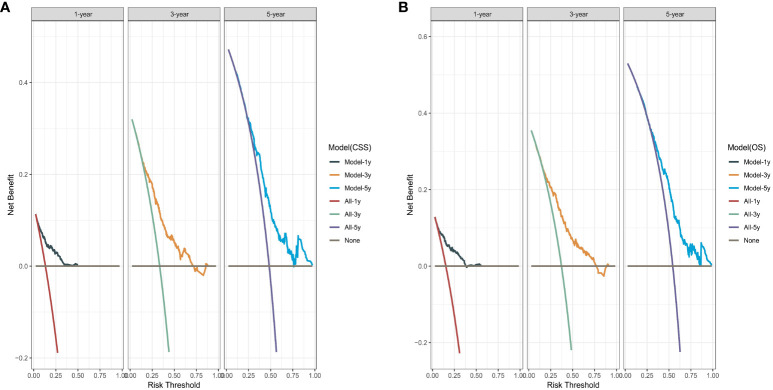
The decision curve analysis. **(A)** Overall survival (OS); **(B)** Cancer-specific survival (CSS).

We performed bootstrapped resample (100 iterations) to validate this nomogram and found that C-index was 0.683 and 0.684 for OS and CSS. The internal calibration curves for 1, 3, and 5-year OS and CSS are shown in **Figure A2**. In addition, the DCA curves show good positive net benefits at 1, 3, and 5 years of OS and CSS (**Figure 7**).

## Discussion

Imatinib is still the first-line regimen for the treatment of advanced and metastatic GIST, limiting the use of surgical resection in GIST liver metastasis (GIST-LM) to some extent (18). However, most GISTs respond to imatinib for 12-36 months, after which more than 80% of patients will develop secondary resistance due to acquired secondary mutations in KIT or PDGFRA that lead to resistance. Although some TKIs are effective against some of these mutations, no single drug has emerged that is effective against all mutations. In addition, in advanced and metastatic GISTs, complete remission is rarely observed after imatinib treatment, and disease progression may occur even after years of treatment (1, 6, 19). Therefore, in theory, surgical resection combined with imatinib therapy seems to be a safe and feasible treatment modality to address this problem, and in recent years, surgical treatment of liver metastatic GISTs has been reported in several studies. Sessing et al. identified a total of 48 patients with liver metastasis GIST who combined surgical resection with imatinib treatment, with 1, 3, and 5-year OS of 93%, 80%, and 76%, respectively, and in multivariate analysis, R0 resection was the only independent significant prognostic factor for DFS and OS (6). Ye et al. conducted a systematic review of articles on GIST liver metastases and found that combining surgery with TKI therapy resulted in R0/R1 resection rates ranging between 48% and 82% across series, which may be an effective treatment for patients with liver metastasis GIST (20). In the National Comprehensive Cancer Network (NCCN) guidelines, lifelong systemic therapy (TKI) is still the first recommended treatment for patients with liver metastatic GIST, but TKI combined with surgery is recommended when liver metastases can be completely resected by an experienced surgeon, and the tumor responds favorably to TKI (21). The prognosis is likely to be worse for patients in whom metastases have already occurred prior to formal initiation of therapy, and salvage therapy is less effective in this population. In addition, the mechanism of resistance to tyrosine kinase inhibition in this population is quite different from that of patients with secondary imatinib resistance, and therefore it remains questionable to provide surgical recommendations for patients with GIST-LMs (18). Some studies have concluded that surgical resection of the primary and liver metastases is feasible, but the importance of complete resection and timing of resection should be noted (22).

In this study, we included GIST patients with only occurring and concurrent liver metastases from 2010-2019 in the seer database, in which TKIs are readily and widely used in this cohort. A 1:1 PSM was performed on the surgical and non-surgical groups, which showed significant improvement in 1, 3, and 5-year OS and CSS in the surgical group compared to the non-surgical group. We also performed further subgroup analysis and found that OS and CSS were significantly better in patients treated with surgery combined with targeted therapy than in patients treated with surgery only, and these results were consistent with other retrospective studies (6, 18, 20, 23–25). Among them, Turley et al. reviewed patients with GIST-LM who underwent hepatic resection at three centers between 1995 and 2010 and found that OS after combination therapy exceeded previous reports of hepatic resection or TKI therapy alone in the treatment of metastatic GIST, and that postoperative TKI therapy significantly improved overall survival (23). This may be due to the reason that surgical resection can eliminate or reduce the tumor load and prolong the duration of tumor resistance, which not only contributes to the possibility of treatment with imatinib as well as other TKIs, but also preserves the possibility of future treatments (1, 25). On the other hand, although surgical treatment is beneficial for patients with GIST-LM, combined resection of the primary tumor and liver metastases may increase the recurrence rate as well as the mortality rate of patients, so the treatment of patients with GIST-LM should be multi-disciplinary, multimodal and comprehensive (23, 26, 27). However, due to the lack of patient data in the Seer database, we were unable to explore this further. Most studies currently consider surgical treatment of patients with liver metastases that respond to TKI therapy and can achieve RO resection of the lesion to be feasible, so the judgment regarding the indication for surgery is of its importance.

In this study, we also explored the factors influencing OS and CSS in GIST-SLM patients and found that age, sex, site, grade, tumor size, lymph node metastasis, chemotherapy, surgery, and marital status were the factors influencing OS and CSS in GIST-SLM patients. Males and age >65 y were significant risk factors for poor OS and CSS. Fero et al. designated GIST patients in the Seer database diagnosed at ages 13-39 years as AYA (adolescent and young adult) and patients aged 40 years or later as OA (old adult) and found that 5-year OS and CSS in the AYA population were significantly higher (OS: 83.3% vs 75.4%, P < 0.001; CSS: 82.4% vs 61.7%, P < 0.001) (28). Rong et al. studied the role of gender in the prognosis of gastric GIST and found that the risk of death was higher in males than in females (HR = 1.677, 95%CI = 1.150-2.444, P = 0.007) (29). Therefore, younger female patients tend to portend a better prognosis, and it has been suggested that male patients tend to have more aggressive GIST, with larger tumors, higher rates of mitotic divisions, and more tumor ruptures and metastases, which could explain the gender difference in CSS (30). However, in this study, this difference was not found due to the completeness of the data, so more studies are still needed in the future to confirm the clinical results and elucidate the underlying pathophysiologic mechanisms.

Tumor size and tumor site are the best-known risk variables for survival and tumor recurrence in GIST, and GIST in non-gastric sites has a worse prognosis than those located in the stomach (28, 31). This is consistent with the results of the present study. However, the risk of small intestinal lesions was not significant compared to intragastric lesions, probably because all GIST-SLM patients were included in this study. Lower OS and CSS in patients with tumor size >10 cm compared to patients with tumor size ≤5 cm has been demonstrated in several studies (1, 15, 16). GIST lymph node metastasis is a rare event, but in the current study, the occurrence of lymph node metastasis tends to portend a poor prognosis, and this effect on survival also correlates with the degree of lymph node burden (32, 33). However, some studies have concluded that lymph node metastasis does not appear to be associated with a poor prognosis (34). The NCCN guidelines do not recommend routine lymph node dissection, but the removal of pathologically enlarged lymph nodes should be considered in patients with known SDH-deficient GIST or GIST known to be associated with translocation (21). In this study, we included all patients with regional lymph node metastasis and included lymph node metastasis as a predictor in the model.

The grade of tumor differentiation is also an important prognostic factor in patients with GIST-SLM. In the results of an analysis of all metastatic GISTs from 2001-2006 in the seer database by Yue et al., patients with well or moderately differentiated tumors had a better OS and CSS than patients with undifferentiated or poorly differentiated tumors (25). The same result was obtained in our study. Marital status also affects survival in GIST-SLM patients. Being unmarried was associated with a significant decline in OS and CSS, which was observed in the vast majority of cancers, and the most vulnerable group was divorced/separated men, highlighting the significant impact that social support may have on malignancy survival (35, 36).

Finally, we integrated the predictive model into a nomogram to quantify the individualized risk of clinical events through simple graphs to guide individualized treatment strategies. In addition, we did not include mitotic count as an influencing factor for OS and CSS, and the effect of high mitotic rate (>5 mitoses/50 HPFs) on OS and CSS was not statistically significant in both univariate and multivariate analyses, which we believe may be related to the following reasons: first, the pathologic grading of mitotic rate in the surgical group may be inaccurate. Neoadjuvant therapy with evidence of pathologic efficacy will not yield accurate mitotic information. In this case, in the absence of mitotic rates, risk stratification may need to be based on clinical parameters, size, and site. Secondly, the proportion of missing values in this variable was as high as 70.4%, including 87.5% in the non-surgical group, which contributed to the instability of this variable.

However, there are limitations to the analysis. First, The PSM method used in the present study does not often achieve complete elimination of correlation as for the retrospective study aiming to control confounders, thereby the presence of selective bias is inevitable. Second, the data in this study came from the Seer database, and some of the variables lacked details, such as the specific type of surgery, the size and number of liver metastases, the detailed chemotherapy regimen and dose, chemotherapy responsiveness, and tumor KIT or PDGFRA mutation information. In this case, it is not possible to conduct an accurate study of treatment options and mutation-related prognosis. Third, the limited sample size and the presence of missing data values may lead to inaccurate models. In view of this, multicenter, large-sample prospective studies are needed to validate the accuracy of the models.

## Conclusion

Our study shows that surgical treatment can improve the OS and CSS of patients with GIST-SLMs. In addition, the combination with chemotherapy may be more favorable for the long-term survival of patients. But it is still necessary to conduct further research on the optimal sequential approach of surgery and chemotherapy. Meanwhile, we identified risk factors affecting the long-term survival of GIST-SLM patients, constructed the nomograms for predicting OS and CSS at 1, 3, and 5-years, and validated them internally. Our model can contribute to clinical management and optimizing treatment strategies.

## Data availability statement

Publicly available datasets were analyzed in this study. This data can be found here: https://seer.cancer.gov/data/access.html.

## Ethics statement

Data in the present research was downloaded from the SEER database of the National Cancer Institute. Ethical approval and informed consent were exempted by the ethics committee owing to the public availability of data in the SEER database. The studies were conducted in accordance with the local legislation and institutional requirements.

## Author contributions

LL: Writing – original draft, Data curation, Formal Analysis, Methodology, Software. XX: Data curation, Writing – original draft. YJ: Software, Writing – original draft. SZ: Writing – original draft. NS: Methodology, Writing – original draft. YD: Writing – review & editing. HZ: Supervision, Writing – review & editing. SL: Conceptualization, Supervision, Writing – review & editing.

## Supplementary material

Supplementary Figure 1 Forest plot of risk factors for survival. **(A)** Overall survival (OS); **(B)** Cancer-specific survival (CSS).

Supplementary Figure 2 The internal calibration curves. **(A)** 1-year OS; **(B)** 3-year OS; **(C)** 5-year OS; **(D)** 1-year CSS; **(E)** 3-year CSS; **(F)** 5-year CSS.
