# Supplementary figures and images for: Effects of surgical management for gastrointestinal stromal tumor patients with liver metastasis on survival outcomes
Source: Front Oncol. 2024 Jan 29;14:1289885. doi: 10.3389/fonc.2024.1289885 (PMC10860711; doi:10.3389/fonc.2024.1289885)

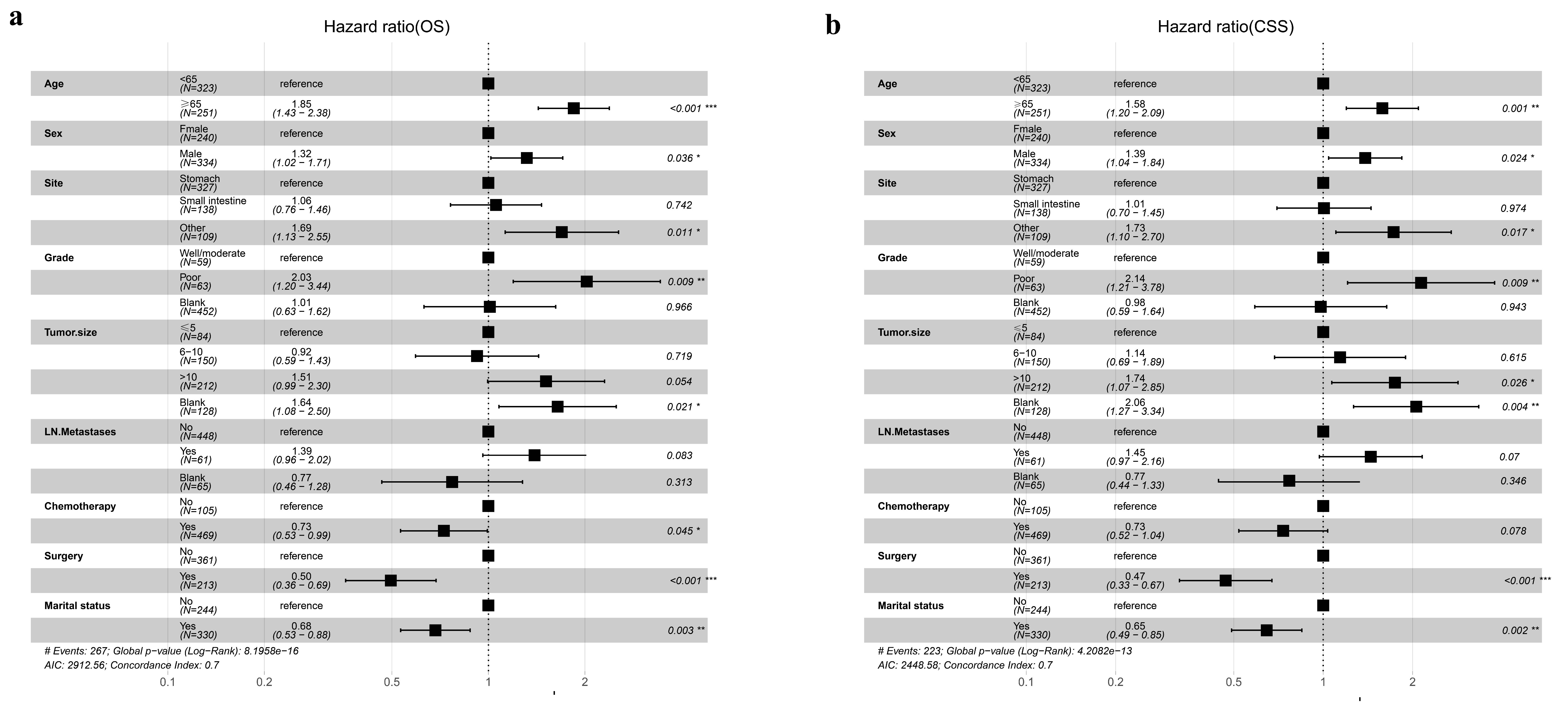

Supplement: Supplementary Figure 1 — Forest plot of risk factors for survival. (A) Overall survival (OS); (B) Cancer-specific survival (CSS). [file Image_1.jpeg]

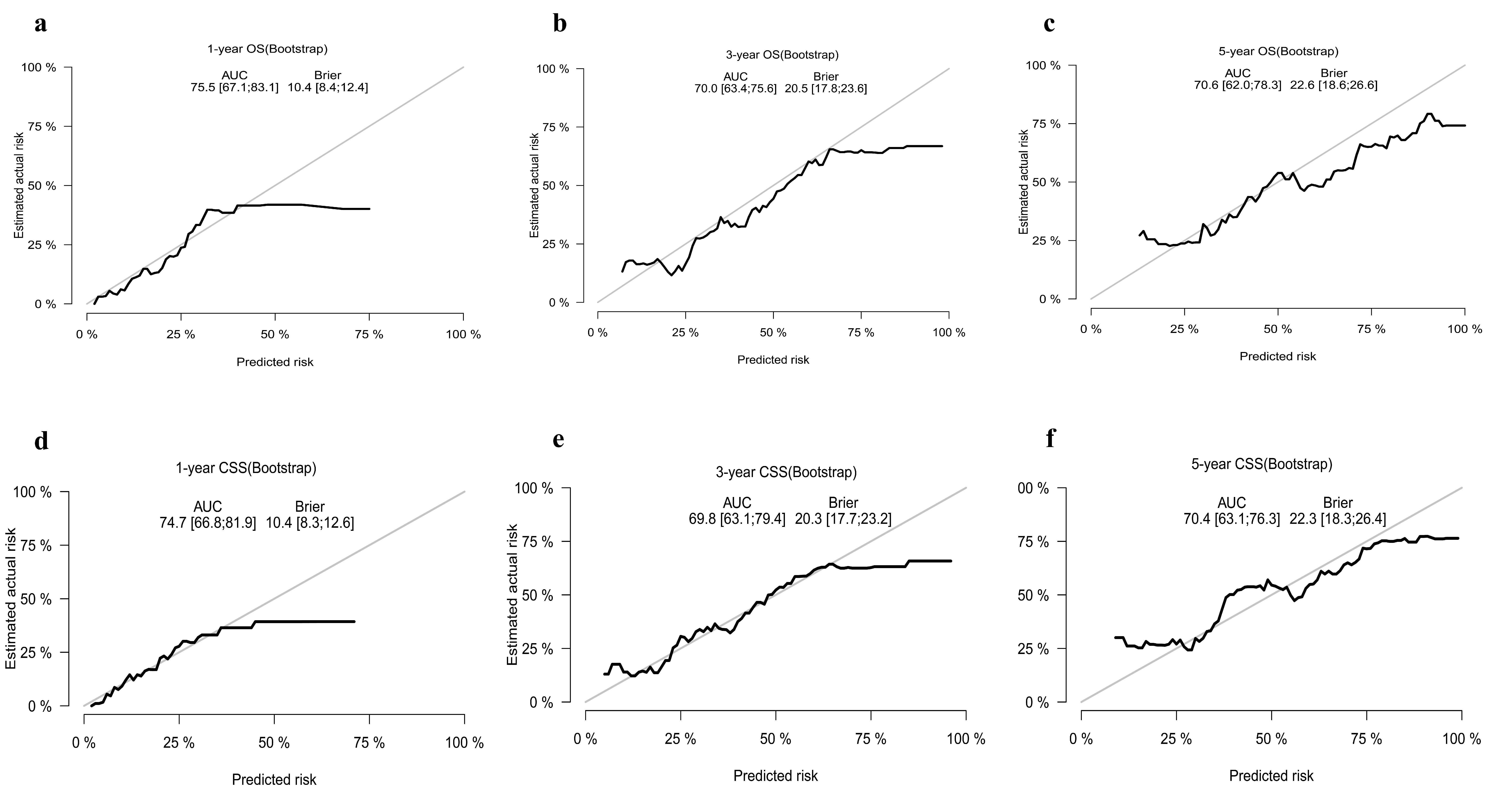

Supplement: Supplementary Figure 2 — The internal calibration curves. (A) 1-year OS; (B) 3-year OS; (C) 5-year OS; (D) 1-year CSS; (E) 3-year CSS; (F) 5-year CSS. [file Image_2.jpeg]
